# Knockdown of Dystrophin Dp71 Impairs PC12 Cells Cycle: Localization in the Spindle and Cytokinesis Structures Implies a Role for Dp71 in Cell Division

**DOI:** 10.1371/journal.pone.0023504

**Published:** 2011-08-19

**Authors:** Marcela Villarreal-Silva, Federico Centeno-Cruz, Rocío Suárez-Sánchez, Efraín Garrido, Bulmaro Cisneros

**Affiliations:** 1 Departamento de Genética y Biología Molecular, Centro de Investigación y de Estudios Avanzados del IPN (CINVESTAV-IPN), México Distrito Federal, México; 2 Laboratorio de Enfermedades Complejas, Instituto Nacional de Medicina Genómica, México Distrito Federal, México; 3 Departamento de Genética, Instituto Nacional de Rehabilitación, México Distrito Federal, México; University of Birmingham, United Kingdom

## Abstract

The function of dystrophin Dp71 in neuronal cells remains to be established. Previously, we revealed the involvement of this protein in both nerve growth factor (NGF)-induced neuronal differentiation and cell adhesion by isolation and characterization of PC12 neuronal cells with depleted levels of Dp71. In this work, a novel phenotype of Dp71-knockdown cells was characterized, which is their delayed growth rate. Cell cycle analyses revealed an altered behavior of Dp71-depleted cells, which consists of a delay in G0/G1 transition and an increase in apoptosis during nocodazole-induced mitotic arrest. Dp71 associates with lamin B1 and β-dystroglycan, proteins involved in aspects of the cell division cycle; therefore, we compared the distribution of Dp71 with that of lamin B1 and β-dystroglycan in PC12 cells at mitosis and cytokinesis by means of immunofluorescence and confocal microscopy analysis. All of these three proteins exhibited a similar immunostaining pattern, localized at mitotic spindle, cleavage furrow, and midbody. It is noteworthy that a drastic decreased staining in mitotic spindle, cleavage furrow, and midbody was observed for both lamin B1 and β-dystroglycan in Dp71-depleted cells. Furthermore, we demonstrated the interaction of Dp71 with lamin B1 in PC12 cells by immunoprecipitation and pull-down assays, and importantly, we revealed that knockdown of Dp71 expression caused a marked reduction in lamin B1 levels and altered localization of the nuclear envelope protein emerin. Our data indicate that Dp71 is a component of the mitotic spindle and cytokinesis multi-protein apparatuses that might modulate the cell division cycle by affecting lamin B1 and β-dystroglycan levels.

## Introduction

Duchenne muscular dystrophy (DMD) is a progressive, X-linked, degenerative muscle disorder caused in the majority of cases by large out-of-frame deletions or duplication in the DMD gene that provoke the absence or dysfunction of the cytoskeletal protein dystrophin [1], [2]. The DMD gene exhibits complex transcriptional regulation; it drives the synthesis of a variety of dystrophin isoforms through utilization of different promoters. Full-length dystrophin (427 kDa) is derived from three independent promoters, located at the 5′-end of the DMD gene, that regulate its spatiotemporal expression in muscles, brain structures, and cell types [3], [4], [5]. In addition, several N-terminally truncated dystrophin variants, named according to their respective molecular weights as Dp260, -116, -140, and -71, are produced from different internal promoters [1], [6].

While dystrophin Dp427 provides integrity to the sarcolemma by connecting the extracellular matrix to the intracellular cytoskeleton [7], Dp71 is thought to be involved in the mental retardation present in one third of patients with DMD because Dp71 is the most abundant DMD gene product in adult brain [1], [8], [9], and, more importantly, because patients with mutations located in the Dp71 coding region that significantly affect Dp71 expression exhibited severe mental retardation [10], [11]. In support of this hypothesis, functional examination of Dp71-null mice revealed abnormal synaptic organization and maturation *in vitro*, reduced synaptic plasticity in CA1 hippocampus, as well as selective behavior disturbances [10]. Thus, it appears that definition of Dp71 function in neuronal cells is a necessary step toward understand the molecular basis underlying DMD-associated mental retardation. Following this direction, we have adopted the PC12 cell line as the model for Dp71 study; these cells have been broadly employed in differentiation studies [12], [13], [14]. In our previous studies, we isolated PC12 cells with depleted Dp71 levels by stable transfection with a vector that expresses an antisense RNA against Dp71 mRNA [15]. Characterization of Dp71-depleted clones provided compelling evidence that Dp71 is crucial for both cell adhesion and nerve growth factor (NGF)-induced neuronal differentiation [15], [16], [17]. Recently, we unveiled a new phenotype in the Dp71-knockdown cells, a marked delay in cell growth, which indicates that Dp71 deficiency might alter the PC12-cell division cycle. In this study, we report, to our knowledge for the first time, the localization of Dp71 in mitotic spindle, cleavage furrow, and midbody. Furthermore, we reveal that Dp71-knockdown cells exhibit altered cell division cycle and provide evidence that such alteration might be caused by the negative effect that Dp71 deficiency exerts on lamin B1 and β-dystroglycan levels.

## Materials and Methods

### Cell culturing

The PC12 cell line [12] was cultured in RPMI-1640 (Invitrogen, Carlsbad, CA, USA) supplemented with 5% (v/v) Fetal bovine serum (FBS), and 10% (v/v) horse serum inactivated at 56°C for 30 min, 100 U/ml penicillin, and 100 µg/ml streptomycin (Invitrogen) and maintained at 37°C in a humidified atmosphere containing 5% CO_2_. Isolation of PC12-derivative clones, termed Dp71-depleted cells and control cells, was described previously [15], [16].

### Proliferation assays

Cells were seeded by triplicate at a density of 2×10^5^/well in six-well plates (Corning 3506, Costar, NY, USA), and the number of viable cells was counted at the indicated times by the trypan blue exclusion method using a Neubauer hemocytometer (Marienfeld-Superior, Germany). Cell viability was also evaluated by the 3-(4,5-dimethylthiazole-2-5-diphenyl tetrazolium bromide (MTT) assay. Cells were harvested during logarithmic growth phase and seeded daily during a period of 6 days in 96-well plates (Corning, Costar) at a density of 1 cell/µL, freshly prepared MTT (Sigma-Aldrich, Inc.) was added to each well to a final concentration of 0.5 mg/ml and plates were further incubated for 4 h at 37°C. Cells were centrifuged for 5 min at 1,500 rpm and the supernatant was discarded; 180 µl of dimethylsulfoxide was then added and plates were shaken until no particulate matter was visible. Absorbance was measured on a Molecular Devices Spectra Max Plus384 microplate reader (Molecular Devices, Sunnyvale, CA, USA) with a test wavelength of 570 nm. The cell growth curve was drawn by plotting the mean cell number of each point against the cell culture time. DNA synthesis was determined by Bromodeoxyuridine (BrdU) incorporation. Cells were seeded in 35×10 mm dishes (Corning, Costar) and cultured for 24 h, pulsed with 10 mM BrdU for 2 h, and fixed to stain with the BrdU flow kit (BD Biosciences Pharmingen, San Diego, CA, USA), accordingly to manufacturer's instructions. Positive BrdU-labeled cells were detected by cytometry in a Becton Dickinson (BD) FACScalibur flow cytometer (BD Biosciences).

### Flow cytometry

Cells were seeded in P-60 dishes (Costar, Corning, NY, U.S.A.) at a density of 1.25×10^5^ cells/ml. After 48 or 72 h of culturing, cells were harvested and cell suspensions were pelleted and washed twice with Phosphate buffered saline (PBS) containing 20% trypsin (Invitrogen) and 0.53 mM EDTA (Research Organics, Cleveland, OH, USA). Cells were then fixed with 80% ethanol for at least 24 h, stained for DNA labeling with 100 µg/ml propidium iodide solution containing 200 µg/ml RNase A, and transferred to flow cytometry tubes for cell cycle analysis in a BD FACScalibur flow cytometer (BD Biosciences). Cell cycle analysis was performed using the ModFit LT software (Verity Software House, Topsham, ME, USA).

### Apoptosis assays

Cells were plated on 35×10 mm dishes (Corning, Costar) at a 1×10^4^ cells/mL density for 48 h. After this time cells were harvested, washed with PBS and stained with the commercial kit annexin-V-FITC Vibrant Apoptosis 2 (Invitrogen, Ca, USA) and 0.5 µg/mL propidium iodide to analyze early and late apoptosis by flow cytometry.

### Cell culture synchronization for cell cycle kinetics analysis

To block cell cycle progression at G0/G1, cells were plated on P100 dishes (Corning, Costar) at a 2×10^6^ density and cultured for 24 h. After this time, the culture medium was replaced with RPMI medium with a low dose of serum (0.05% FBS and 0.20% horse serum) and cells were cultured for additional 12 h. Cells were released from G0/G1 by reconstituting the culture medium with normal sera concentrations. Then, cells were harvested at 0, 12, 16, 19, and 23 h for further analysis. To perform the double-block of cells at S phase, cells were plated and incubated for 24 h in normal conditions, afterward, thymidine was added to the culture medium at a final concentration of 2 mM and cells were incubated for additional 24 h, then collected, washed with PBS, and incubated again for 12 h in fresh culture medium. After this time, thymidine was again added to the culture medium and cells were incubated for a final period of 16 h. To release cells from S phase arrest, culture medium was replaced by fresh medium. To block cell cycle progression at M phase, cells were cultured for 24 h in 35×10 mm dishes, and then nocodazole was added to the culture medium to final concentrations of 0.2, 0.4, and 0.7 µg/ml (Sigma-Aldrich, Inc.). After 36 h of exposure, cells were collected, washed, resuspended in PBS, and attached to glass coverslips by centrifugation for 5 min at 350 rpm in a Cytospin 4 Cytocentrifuge (Thermo Fisher Scientific. Inc., Waltham, MA, USA) and fixed to perform immunofluorescence analysis. In all cases, cell cycle analysis was performed after cell fixation and PI-staining.

### Cell culture synchronization for cytokinesis analysis

To analyze cytokinesis by immunofluorescence, we blocked cell cycle progression at the S phase by incubating cells with 2 mM thymidine (Sigma-Aldrich, Inc.) for 24 h. Cells were released from arrest by washing with PBS and then incubated for 8–9 h in fresh culture medium on glass coverslips.

### Immunofluorescence and confocal microscopy analysis

Cells cultured on glass coverslips were washed with PBS, fixed with 4% paraformaldehyde in PBS for 10 min at room temperature, permeabilized with 0.2% triton X-100 in PBS for 5 min at 4°C, blocked with 0.5% gelatin and 1.5% FBS in PBS for 20 min at room temperature, and incubated overnight at 4°C with the appropriate primary antibody. Next, coverslips were incubated for 1 h with either fluoroisothiocyanate (FITC) labeled goat anti-rabbit secondary antibody (Zymed Laboratories, Inc., South San Francisco, CA, USA), tetramethylrhodamine isothiocyanate (TRITC) labeled goat anti-mouse secondary antibody (Jackson Immunoresearch Laboratories, West Grove, PA), or AlexaFluor594-labeled chicken anti-goat secondary antibody (A21468, from Invitrogen). For counterstaining, cells were incubated for 10 min at 37°C with 1 µg/ml 4′,6-diamidino-2-phenylindole (DAPI) in PBS. After washing, coverslips were mounted on microscope slides with VectaShield (Vector Laboratories, Inc., Burlingame, CA, USA) and analyzed in a confocal and multiphoton microscope (TCS-SP5, Leica Microsystems, Heidelberg, Germany), using an oil immersion 63× objective. Co-localization of FITC, TRITC, and DAPI staining was analyzed in single optical sections obtained for two channels throughout the Z axis. To analyze mitosis by immunofluorescence and confocal microcopy analysis, cells were blocked at the S phase with thymidine, as described previously, then released from arrest by washing with PBS and platting in fresh culture medium on glass coverslips for 8–9 h (metaphase-anaphase) or 10 h (cytokinesis), and stained with PI.

### Antibodies

Three different rabbit polyclonal pan-dystrophins antibodies, directed against the same epitope at the C-terminal domain of Dp71, were used: +78 (Genemed Synthesis, Inc., San Francisco, CA, USA) [18], 2166 [19], and H4 [20]. Based on the suitability of these antibodies for an specific application: 2166 antibody was used solely for western blotting, while +78 and H4 antibodies were employed exclusively for immunofluorescence and immunoprecipitation, respectively. In addition, the following antibodies were employed: a monoclonal anti-actin antibody [21]; rabbit polyclonal antibodies against lamin A/C (H-110), emerin (FL-254), cycline B1 (H-433), and anti-actin (H-196); mouse monoclonal anti-α-tubulin antibody (B-7), and a goat anti-β-dystroglycan (DgC20) antibody (Santa Cruz Biotechnology, Inc., CA, USA); a mouse monoclonal anti-lamin B1 antibody (Zymed Laboratories, CA, USA); a rabbit polyclonal anti-lamin B1 antibody (ab16048, Abcam, Cambridge, UK); a mouse monoclonal anti-dystrophin antibody (Mandra 1, Sigma-Aldrich, St. Louis, MO, USA), and a rabbit polyclonal anti-β-dystroglycan antibody (LG5) that recognizes the last seven amino acids of the C-terminal of β-dystroglycan [20].

### Isolation of cell extracts

To obtain whole cell extracts, cultured cells were collected, washed twice with PBS, and centrifuged at 1,200 rpm for 5 min. Pellets were resuspended in a sonication buffer (1× protease inhibitor cocktail -Complete, Roche Applied Science, Indianapolis, IN, USA- containing 10 mM dithiothreitol (DTT) and 1 mM phenylmethylsulphonyl fluoride (PMSF) and sonicated four times for 15 sec at 3.5 µm. To obtain nuclear extracts, pellets were resuspended in cold buffer I (0.32 M sucrose, 3 mM calcium chloride, 2 mM magnesium acetate, 0.1 mM ethylenediaminetetraacetic acid (EDTA), 10 mM Tris.HCl [pH 8.0], 1 mM DTT, 0.5 mM PMSF, and 0.5% Nonidet P-40) and then homogenized with a glass Dounce homogenizer. Suspension was then centrifuged at 1,200 rpm for 10 min to separate nuclei from cytoplasmic fraction (supernatant). Nuclei were resuspended in buffer II (1.2 M sucrose, 3.5 mM magnesium acetate, 0.1 mM EDTA, 10 mM Tris.HCl [pH 8.0], 1 mM DTT, and 0.5 mM PMSF) and subjected to ultracentrifugation at 16 rpm for 45 min through a 2 M sucrose cushion. Purified nuclei were either stained with Propidium iodide (PI) and fixed for cytometry analysis or sonicated in sonication buffer for western blot analysis. To obtain nuclear matrix extracts, purified nuclei were incubated for 15 minutes at 4°C in a 0.5% Triton X-100 solution (with 10 mM Tris-HCl pH 7.4 and 2.5 mM MgCl_2_), then centrifuged at 5000 g for 10 minutes. Nuclear pellet was incubated for 2 hours at 37°C in a 250 U/ml DNAse I solution (with 10 mM Tris-HCl pH 7.4, 150 mM NaCl and 5 mM MgCl_2_) in constant rotation, then an equal volume of a solution containing 10 mM Tris-HCl pH 7.4 and 1.3 M (NH_4_)SO_4_ was added and nuclear suspension was incubated for 15 minutes at 4°C. Afterward, this nuclear suspension was centrifuged at 10000 g for 10 minutes and the nuclear matrix pellet was sonicated as above. Protein concentrations were determined by the Bradford method.

### Western blot analysis

Equal amounts of whole cell protein extracts (60 µg) were mixed with Tris-glycine sodium dodecyl sulphate (SDS) sample buffer and proteins were denatured by boiling for 3 min. Lysates were then separated by 10% SDS-PAGE and electrotransferred to nitrocellulose membranes. Membranes were incubated for 1 h in TBS-T (150 mM NaCl, 10 mM Tris-HCl, pH 8, 0.05% Tween 20) containing 6% low-fat dried milk and then incubated overnight with the corresponding primary antibody. After three washes with TBS-T, membranes were incubated with the appropriate horseradish peroxidase-conjugated secondary antibody (Amersham-Pharmacia, GE Healthcare, Buckinghamshire, UK) and developed using the ECL Western blotting analysis system (Amersham-Pharmacia).

### Immunoprecipitation

Total protein extracts in a final volume of 250 µl were incubated for 1 h at 4°C with 5 µg of anti-lamin B1 antibody, previously bound to protein G-agarose (Invitrogen). As negative control, parallel incubation with an irrelevant rabbit polyclonal antibody bound to protein G-agarose was performed. The immune complexes were precipitated by centrifuging for 2 min at 2,500 rpm and washed twice in RIPA buffer (160 mM NaCl, 10 mM Tris-HCl (pH 7.5), 1 mM EDTA, 1 mM egtazic acid (EGTA), 20 mM Na_3_MoO_4_, 20 mM NaF, 2 mM NaVO_4_, 1 mM PMSF) containing complete protease inhibitor mixture (Roche Applied Science). Precipitated proteins were separated by SDS-PAGE and analyzed by Western blotting.

### Construction of the glutathione-S-transferase (GST)-Dp71 fusion protein

The human Dp71 cDNA was amplificated from pGFP-Dp71 and cloned in the EcoRI site in-frame to the 3′-end of GST in the bacterial expression vector pGex-4T1 (Amersham Biosciences Co., Piscataway, NJ, USA) to generate GST-Dp71 gene fusion. To express and characterize GST and GST-Dp71 fusion protein, an aliquot (40 ml) of transformed bacterial cells (strain JM109) induced with 0.3 mM IPTG for 1 h at 25°C was centrifuged at 10,000 rpm for 10 min, resuspended in 1 ml of NETN buffer [100 mM (w/v) NaCl, 20 mM (w/v) Tris-HCl (pH 7.5), 1 mM (w/v) EDTA, 0.5% (v/v) NP40, 1 mM (w/v) PMSF, complete protease inhibitor cocktail], and sonicated on ice with 4 pulses of 15 sec in a sonicator Soniprep 150-Sanyo. Afterward, 100 µl of packed glutathione-Sepharose 4B beads (Amersham Biosciences Co) were added to bacterial lysate and the mixture was incubated overnight at 4°C with constant rotation. Beads, recovered by centrifugation at 3,000 rpm for 5 min, were washed three times with 1 ml of ice-cold NETN buffer and resuspended in 100 µl buffer NETN. GST and GST-Dp71 proteins, bound to glutathione-Sepharose, were eluted by adding an equal volume of 3× sample buffer and heating at 95°C and subsequently analyzed by Coomassie brilliant blue staining and immunoblotting.

### Pull-down assays

A similar amount of GST or GST-Dp71 fusion protein immobilized onto 20 µl of glutathione-Sepharose beads was incubated overnight at 4°C on a rotator with 1 mg of cell extract prepared in RIPA buffer containing 1% (v/v) Triton X-100. Beads were recovered by centrifugation at 3,000 rpm for 5 min and washed three times with 1 ml ice-cold RIPA buffer containing 1% (v/v) Triton X-100. Finally, glutathione-Sepharose-bound proteins were eluted by adding an equal volume of 3× sample buffer, heating at 95°C, and were subsequently analyzed by SDS-PAGE and immunoblotting.

## Results

### Dp71-depleted Cells Display Altered Proliferation

**Figure 1:**
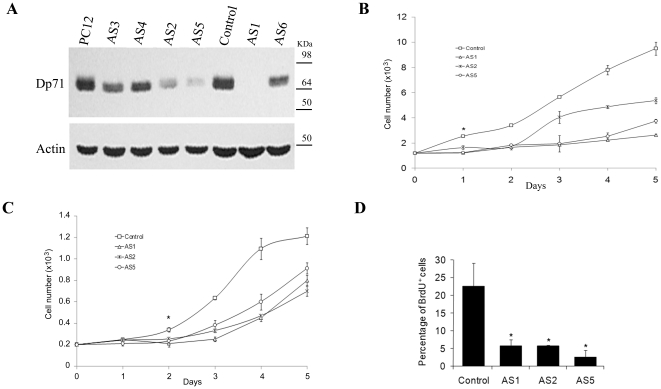
Knockdown of Dp71 expression decreases the proliferation of PC12 cells. Dp71 protein levels were measured by western blotting in different Dp71-depleted clones (AS1–AS6) with the anti-Dp71 antibody 2166 and compared with those of PC12 cells and control cells (control). Membranes were stripped and reprobed with an anti-actin antibody for normalization. Migration of protein markers are denoted on the right (A). Cell proliferation of control and AS1, -2, and -5 cells was monitored over a 5-day period by both direct counting of viable cells via tripan blue exclusion (B) or MTT assay (C) and after 2 days of culturing, by BrdU incorporation using flow cytometry (D). Data are expressed as mean ± standard error of mean (SEM) of three independent experiments. Asterisks in Panel D denote significant differences (*p*<0.05).

During routine culturing of Dp71-depleted cells, we observed a marked delay in cell growth compared with PC12 cells (untransfected) and control (PC12 cells stably transfected with empty vector) clones. After numerous cell passages, Dp71-depleted clones reverted their phenotype and recovered both typical proliferation of PC12 cells and normal expression of Dp71 (data not shown), which suggests a link between the presence of Dp71 and PC12 cell growth. To analyze this phenomenon in depth, unfrozen fresh cultures of six different Dp71-knockdown clones (AS1-AS6) were analyzed for Dp71 expression and cell proliferation. Figure 1A shows that AS1 exhibited maximal depletion of Dp71 protein levels followed by AS5 and -2, compared with PC12 cells and control cells. Therefore, the effect of Dp71 depletion on cell growth was determined in AS1, -5, and AS2 (the clones with lowest Dp71 protein levels) by hemocytometer counting, MTT assay, and BrDU incorporation. All Dp71-depeleted clones tested exhibited a significant decrease in their growth rates from day 2 of culture (*p* = 0.05), peaking at day 5 with reductions of 42–72% and 24–42% by hemocytometer counting (Figure 1B) and MTT assay (Figure 1C), respectively. In concordance, these mutant clones displayed a significant decrease of 80–90% in the percentage of BrdU-positive cells (Figure 1D) compared with control cells. To ascertain whether reduced rates of cell growth are a consequence of cell death due to necrosis or apoptosis, AS1, -2, and -5 clones were analyzed by both trypan blue exclusion and annexin V-staining assays. Figure S1 shows that the percentage of cell death and apoptosis was similar between control and AS1 clone (the most affected Dp71-knockdown clone); thus, impaired proliferation of Dp71-knockdown cells suggested a possible alteration in the cell cycle of PC12 cells due to depletion of Dp71.

### Effect of Dp71-knockdown on Cell Cycle Progression

**Figure 2:**
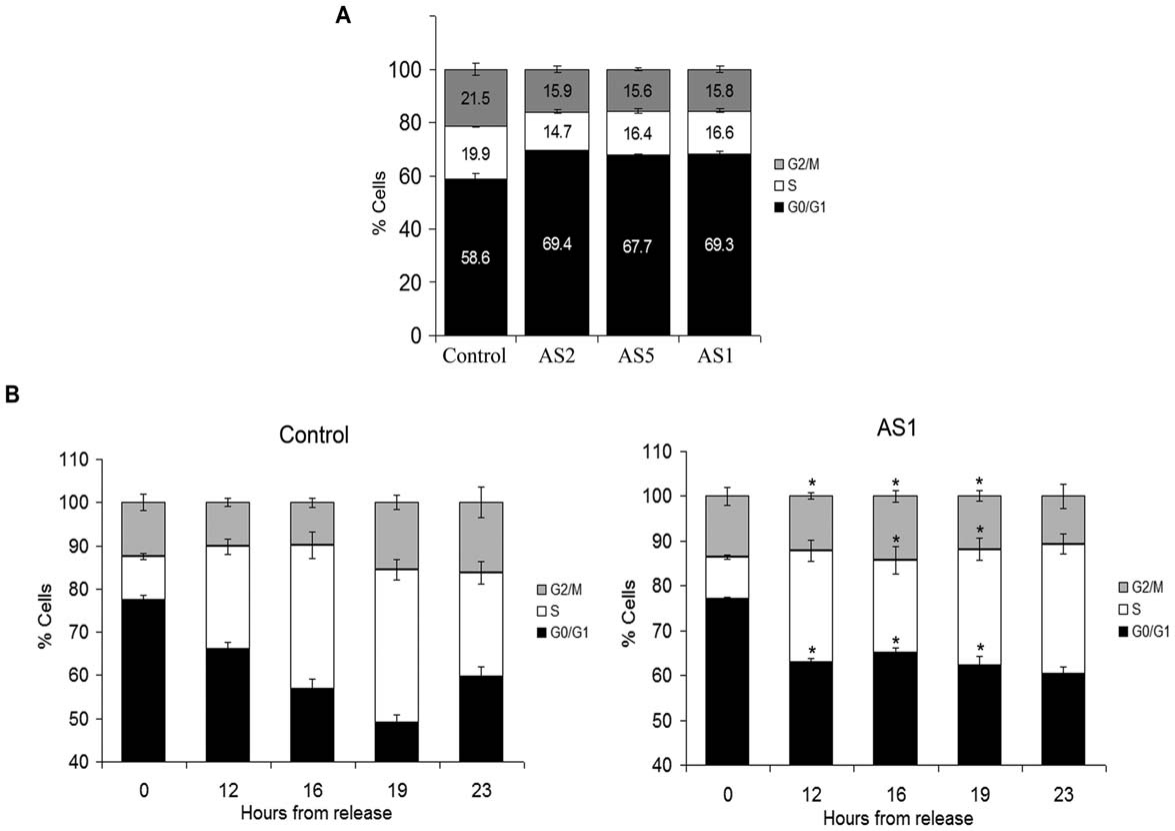
Dp71-depleted cells display altered cell cycle profile and delayed G1-S transition. Control and Dp71-knockdown cells (clones AS1, -2, and -5), cultured for 48 h, were fixed and stained with propidium iodide. DNA content was analyzed by flow cytometry to obtain cell percentages at each cell cycle phase with the use of ModFif software (A). Control and AS1 cell cultures were synchronized at G0/G1 cell cycle phase by serum starvation for 48 h and then released into the cell cycle. At the indicated times, the cells were fixed and progress of the cell division cycle was analyzed by flow cytometry (B). Cell content (%) at each cell cycle phase is shown. Data are expressed as mean ± standard error of mean (SEM) of three independent experiments. Asterisks denote significant differences (*p*<0.05) between the respective phases of AS1 and control cells.

To determine whether decreased proliferation of Dp71-depleted cells is caused by a primary defect in cell cycle, we analyzed cell cycle progression in asynchronic cultures of control and Dp71-knockdown clones (AS1, -2, and -5) by Flow cytometric assays (FACS). Cell cycle profiles of all Dp71-depleted clones tested exhibited a subtle but reproducible increase of ∼10% in G0/G1 phase and concomitant decreases in S (4–6%) and G2/M (5%) phases relative to control cells (Figure 2A). The absence of pronounced steady-state changes in the cell cycle profile of mutant cells would not preclude differences in the transition rate through cell cycle checkpoints. Then, to analyze the effect of Dp71 depletion on G0/G1 phases specifically, control and AS1 cell cultures were blocked at G0/G1 by serum starvation and then released into the cell cycle by adding serum. At 12 h post-release, both cell cultures initiated transition from GO/G1 to S; however, while control cells continued to shift toward S phase at up to 19 h post-release, Dp71-depleted cells stopped moving from G0/G1 to S from 12 h post-release (Figure 2B). At 13 h post-release control cells started to pass from S to G2/M as well as from G2/M to G0/G1, while a small fraction of Dp71-knockdown cells seems to transit from G0/G1 to S. This data suggests that Dp71-depleted cells have a delayed transition from G0/G1 to S or alternatively, a pool of this clone is unable to exit G0/G1. Experiments of S phase arrest with double treatment with thymidine and further release into cell cycle by thymidine withdrawal evidenced that S-G2/M transition of Dp71-knockdown cells is not defective; in fact, progress from S to G2/M appeared to occur faster in Dp71-depleted cells than in control cells (8 vs. 12 h) (Figure S2).

**Figure 3:**
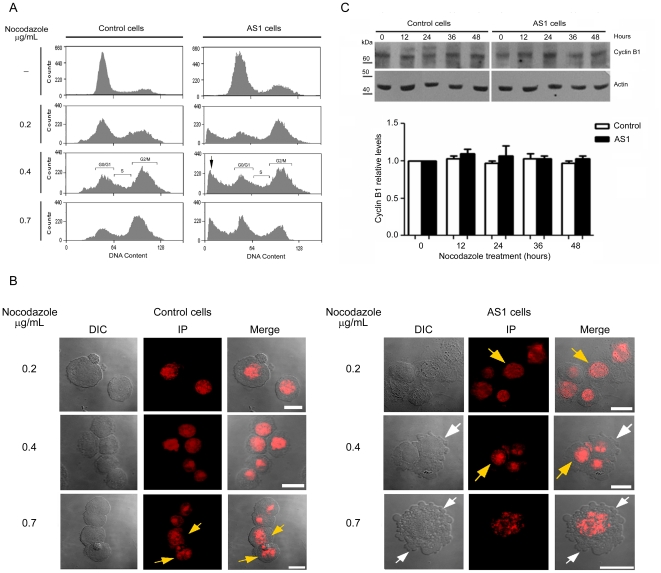
Induction of apoptosis by nocodazole exposure of Dp71-depleted cells. (A) Asynchronous cultures from control and Dp71-knockdown cells (AS1) were incubated for 36 h in normal conditions (-) or with nocodazole at the indicated concentrations. Then, cells were fixed and stained with propidium iodine for flow cytometry analysis. Sub-G0 cell population in AS1cells is denoted by an arrow. (B) Nocodazole-treated control and AS1 cells were stained with propidium iodide, adhered to coverslips, and analyzed by fluorescence confocal microscopy. DIC, differential interphase contrast. Merged images are shown on right panels. White arrows point to membrane protrusions of apoptotic cells and yellow arrows to DNA arrangement in mitotic-arrested cells. Bar = 10 µm. (C) Total protein extracts obtained from control and Dp71-depleted cells treated with 0.2 µg/mL nocodazole were analyzed by western blotting with an anti-cycline B1 antibody. Membranes were stripped and reprobed with an anti-actin antibody for normalization. Migration of protein markers are denoted on the right. Relative protein levels of cycline B1 in untreated control cells were set at 1. Results are expressed as mean ± Standard deviation (SD) of three independent experiments. Asterisk denotes significant differences (*p*<0.05). Data are expressed as mean ± standard error of mean (SEM) of three independent experiments.

Next, control and Dp71-knockdown cells were exposed to different concentrations of nocodazole (0.2, 0.4, and 0.7 µg/ml) for 36 h to induce mitotic arrest. Flow cytometric analysis showed that control cells were arrested efficiently at G2/M with 0.7 µg/ml of nocodazole, while Dp71-depleted cells exhibited a biphasic, dose-response behavior, with mitotic arrest at low drug concentrations (0.2 µg/ml) and resistance to G2/M block at higher drug concentrations (0.7 µg/ml) (Figure 3A). It is noteworthy that Dp71-knockdown cells treated with 0.4 or 0.7 µg/ml of nocodazole showed a prominent peak at sub-G0/G1 that might correspond with cells undergoing apoptosis (Figure 3A). To address this possibility, the morphology of control and Dp71-depleted cells exposed to nocodazole was analyzed by confocal microscopy. Control cells treated with the highest concentration of nocodazole (0.7 µg/ml), as well as Dp71-depleted cells treated with the lowest concentration of nocodazole (0.2 µg/ml), displayed the classical morphology of cells at mitotic prophase, evidenced by increased nuclear size and chromosome condensation (yellow arrows), while the Dp71-depleted cells treated with higher drug concentrations (0.4 and 0.7 µg/ml) exhibited morphological alterations that are consistent with apoptosis, including cell shrinkage, pyknosis, extensive plasma membrane blebbing, and karyorrhesis (white arrows) (Figure 3B). The fate of cells arrested at mitosis is decided by two opposite networks, one that involves activation of the caspase-dependent cell death pathway, and another that controls exit of mitosis through degradation of cyclin B1 (mitotic slippage) [22], [23], [24], Therefore, the expression of cycline B1 was evaluated in Dp71-depleted cells at 0, 12, 24, 36 and 48 hrs of nocodazole treatment (0.2 µg/ml). Figure 3C shows that cycline B1 levels remained constant during nocodazole-induced mitotic arrest in both control and Dp71-depleted cells, which supports the hypothesis that Dp71-depleted cells undergo mitotic death.

The abnormal behavior displayed by Dp71-knockdown cells in response to nocodazole treatment precluded comparative analysis of cell cycle progression from G2/M between control and mutant cells, but evidenced that Dp71-depleted cells might have altered mitosis.

### Distribution of Dp71 in Control and Dp71-knockdown Cells in Mitosis and Cytokinesis

**Figure 4:**
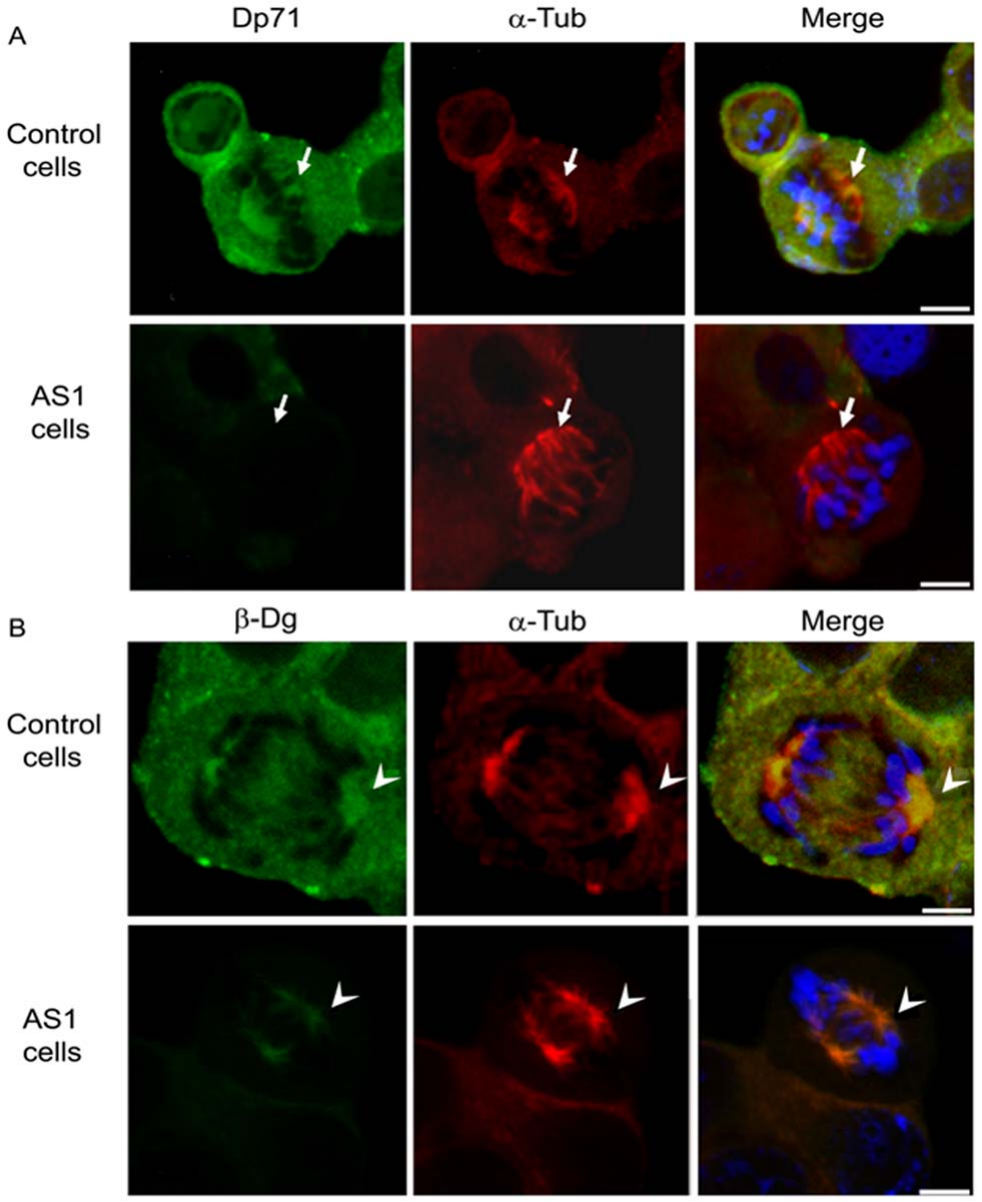
Dp71 and β-dystroglycan localization in control and Dp71-knockdown cells in mitosis. Control and Dp71-depleted (clone AS1) cells were arrested with thymidine for 24 h and released by culturing for 10 h on glass coverslips with fresh culture medium. Cells were double stained with antibodies against Dp71 (+78 antibody) and α-tubulin (A) or β-dystroglycan (β-Dg, LG5 antibody) and α-tubulin (B). The specific protein signals were developed using the appropriate FITC- or TRITC-conjugated secondary antibodies. Cells were counterstained with DAPI (blue color) to visualize nuclei. After labeling, cell preparations were subjected to confocal microscopy analysis, and single optical Z-sections were selected to show the distribution of each protein. Merged images are shown in right panels. Arrows and arrow heads denote the presence of Dp71 and β-dystroglycan in the mitotic spindle poles, respectively. Bar = 5 µm.

**Figure 5:**
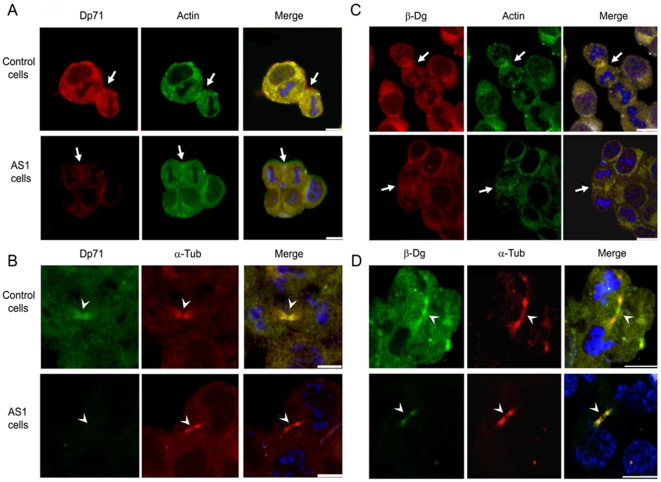
Targeting of Dp71 and β-dystroglycan to cleavage furrow and midbody in control and Dp71-depleted cells in cytokinesis. Control and Dp71-antisense (AS1) cells seeded on coverslips were arrested at S phase with thymidine for 24 h and released by culturing for 10 h with fresh culture medium. Cells were double stained for Dp71 (Mandra 1 antibody) and actin (H-196) (Panel A), Dp71 (+78) and α-tubulin (B-7) (Panel B), β-dystroglycan (β-Dg, DgC20 antibody) and actin (H-196) (Panel C), or β-dystroglycan (DgC20) and α-tubulin (B-7) (Panel D). The specific signal was developed using the appropriate FITC- or TRITC-conjugated secondary antibodies. Cells were counterstained with DAPI (blue color) to visualize nuclei and subjected to confocal microscopy analysis. Single optical Z-sections were selected to show the distribution of each protein. Merged images are shown in right panels Arrows point to co-localization of Dp71 or β-dystroglycan with actin at the cleavage furrow (Panels A and C respectively), whereas arrow heads point to co-localization of Dp71 or β-dystroglycan with α-tubulin at the midbody (Panels B and D respectively). Bar = 5 µm.

As a first step to ascertain whether Dp71 might possess a functional contribution to mitosis, immunofluorescence localization of Dp71 was monitored in control and Dp71-depleted cells that were previously released to cell division from a thymidine-induced S phase arrest. Cell preparations were co-stained with either α-tubulin to detect mitotic spindles and midbody in cells undergoing metaphase-anaphase or cytokinesis, or actin, to decorate the cleavage furrow in cells in cytokinesis. Because β-dystroglycan, a well-characterized Dp71-associated protein, is thought to be involved in cell cycle modulation and has been localized to cleavage furrow and midbody in cytokinesis [25], its spatial distribution was compared with that of Dp71. Confocal microscopy analysis showed that Dp71 was localized throughout the cell body of control cells, but with greatest intensity at metaphase mitotic spindle and poles, where it co-localized with α-tubulin (Figure 4A, upper panels). As expected, Dp71 immunostaining was virtually absent in mitotic spindles of Dp71-depleted cells (Figure 4A, lower panels). Immunolabeling of β-dystroglycan and α-tubulin co-localized to mitotic spindle of control cells, accumulating to the greatest degree at polar regions of mitotic spindle (Figure 4B, upper panels). Interestingly, β-dystroglycan immunostaining exhibited a marked reduction in whole body of Dp71-depleted cells, but continued to be enriched to a certain extent in mitotic spindle poles, which suggests that secondary interactions with mitotic spindle proteins may partially stabilize β-dystroglycan in the absence of Dp71 (Figure 4B, lower panels). With respect to cytokinesis of control cells, Dp71 labeling became enriched in cleavage furrow (Figure 5A, upper panels) and with greatest intensity in midbody (Figure 5B, upper panels) of dividing cells, co-localizing with actin and α-tubulin respectively. In contrast, Dp71-knockdown cells displayed a faint or almost undetectable signal for Dp71 in cleavage furrow (Figure 5A, lower panels) and midbody (Figure 5B, lower panels). Likewise, β-dystroglycan staining accumulated in cleavage furrow (Figure 5C, upper panels) and midbody (Figure 5D, upper panels) of control cells in division, but to a lesser extent in Dp71-depleted cells (Figures 5C and D respectively, lower panels).

### Knockdown of Dp71 Expression Decreases Lamin B1 Levels

**Figure 6:**
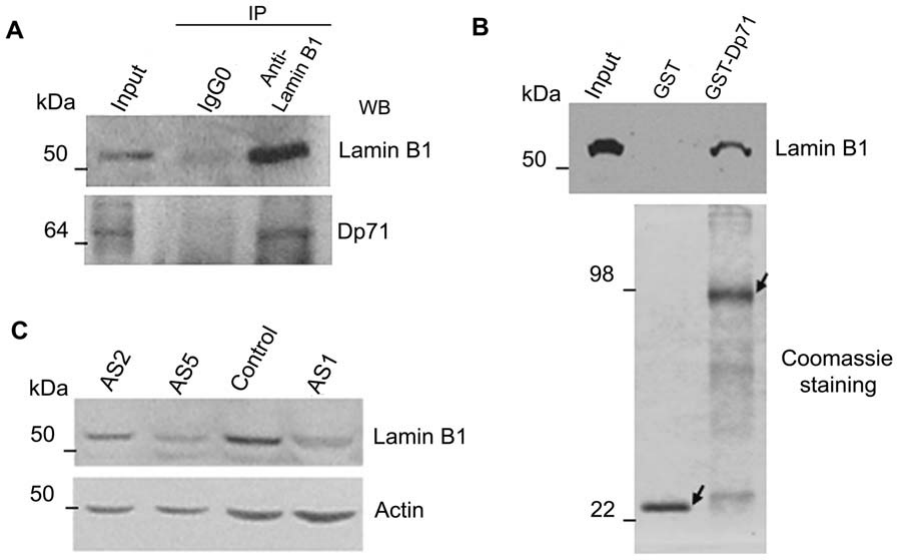
Dp71 associates with lamin B1 in PC12 cells and Dp71-depleted cells display decrease lamin B1 levels. Nuclear extracts from control cells were immunoprecipitated with an anti-lamin B1 antibody, or an irrelevant antibody (IgG0) as control. Immunoprecipitated proteins were analyzed by western blotting with anti-lamin B1 and anti-Dp71 (H4) antibodies (A). GST and GST-Dp71 fusion proteins were expressed in a bacterial system, purified by incubation of bacterial lysates with glutathione beads, and visualized by SDS-PAGE followed by Coomassie brilliant blue staining. GST and GST-Dp71 proteins were immobilized on glutathione-Sepharose beads and incubated with PC12 nuclear extracts to perform affinity pull-down assay (B). Total cell extracts from control and Dp71-depleted (AS1, -2, and -5 clones) cells were resolved by SDS-PAGE and subjected to western blotting analysis with anti-lamin B1 antibody. Membranes were stripped and reproved with an anti-actin antibody for normalization (C). Migration of protein standards are indicated on the left.

As lamin B1 is critical for mitosis, functioning in spindle assembly [26], chromosome segregation, and post-mitotic nuclear assembly [27] and was previously found associated with Dp71 in HeLa cell nuclei at our laboratory [28], we hypothesized that participation of Dp71 in mitosis might take place via its interaction with lamin B1. To approach this hypothesis, as a first step, potential interaction between Dp71 and lamin B1 in PC12 cells was evaluated. Total protein extracts from PC12 cells were immunoprecipitated with an anti-lamin B1 antibody and precipitated proteins were analyzed by immunoblotting with antibodies directed specifically to either lamin B1 or Dp71. Figure 6A shows that lamin B1 was pull-down together with Dp71 by the anti-lamin B1 antibody, whereas none of these two proteins was recovered when an irrelevant antibody (IgG0) was used for immunoprecipitation, establishing the specificity of the assays. To test whether Dp71 directly binds to lamin B1 *in vitro*, pull-down assays were carried out employing GST-Dp71 fusion protein as affinity matrix and PC12 nuclear extracts. GST (negative control) and GST-Dp71 proteins, expressed and purified from JM109 bacterial cultures, were immobilized on glutathione-Sepharose beads and incubated with PC12 nuclear extracts. Nuclear proteins that bound specifically to GST or GST-Dp71 were eluted and analyzed by immunoblotting with anti-lamin B1 antibody. As shown in Figure 6B, lamin B1 was found associated with GST-Dp71, but not with GST alone.

To ascertain whether the interaction of Dp71 with lamin B1 is functionally relevant, we analyzed whether or not the knockdown of Dp71 alters lamin B1 protein levels. Hence, total protein extracts from control cells and Dp71-knockdown cells (AS1, -2, and -5 clones) were analyzed by Western blotting with antibodies raised against lamin B1 and actin (loading control). It is noteworthy that a drastic reduction in the lamin B1 protein band was observed in all Dp71-depleted clones tested compared with control cells (Figure 6C).

Given the negative effect of Dp71 depletion on lamin B1, we were prompted to analyze the protein levels of lamin A/C and emerin, (nuclear envelope proteins functionally related with lamin B1) in Dp71-knockdown cells. Control and Dp71-depleted cells were fractionated into total nuclear and cytoplasmic extracts for Western blot analysis utilizing antibodies against lamin A/C, emerin, and actin (loading control). It is noteworthy that while lamin A/C exhibited exclusive nuclear distribution in both control and Dp71-depleted cells, emerin displayed aberrant cytoplasmic distribution in Dp71-knockdown cells compared with control cells (Figure S3).

### Decreased Lamin B1 Labeling in Dp71-depleted Cells in Mitosis and Cytokinesis

**Figure 7:**
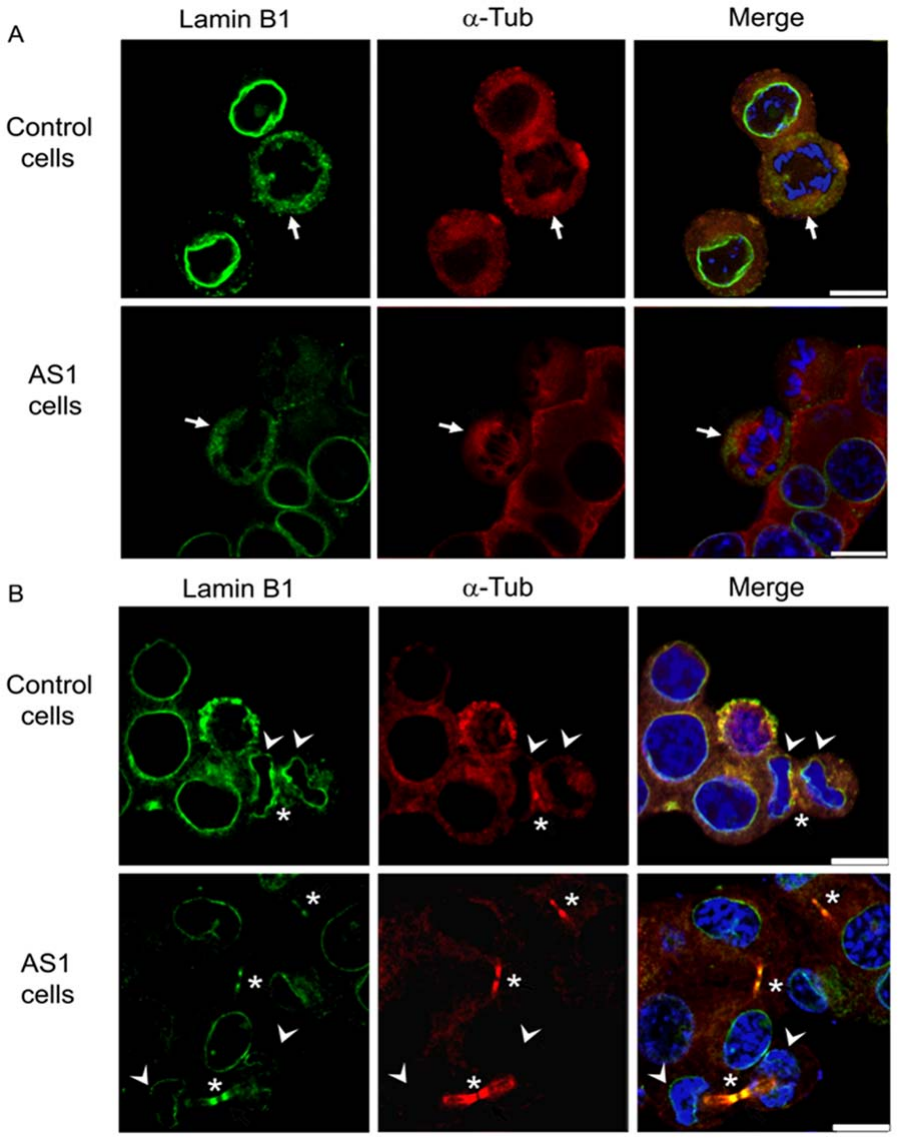
Decreased immunolabeling of lamin B1 in Dp71-depleted cells at mitosis and cytokinesis. Distribution of lamin B1 during interphase and mitosis (A) and during cytokinesis (B) was analyzed in control and Dp71-knockdown (AS1) cells released for 8 and 10 h from a thymidine-induced arrest. Cell preparations were double stained with anti-lamin B1 and anti-α-tubulin antibodies and counterstained with DAPI (blue color) for confocal microscopy analysis. Merging of images is shown in right panels. Arrows, arrow heads and asterisks point to lamin B1 localization at mitotic spindle, reforming nuclei and midbody respectively. Bar = 10 µm.

The spatial distribution of lamin B1 during mitosis and cytokinesis was investigated by immunofluorescence and confocal microscopy analysis in control and Dp71-knockdown cells, which were previously released from thymidine-induced S phase arrest. Co-staining of α-tubulin was employed to observe mitotic spindle and midbody in cytokinesis. As can be seen in Figure 7A (upper panels), immunolabeling of lamin B1 decorated the nuclear envelope in control cells at interphase, while in early mitosis (metaphase), its staining becomes diffuse due to nuclear disintegration, and co-localized with mitotic spindles at the chromosomes periphery (arrow). In Dp71-depleted cells, immunolabeling of lamin B1 was markedly reduced at the nuclear envelope in interphase as well as mitotic spindle poles and peripheral region surrounding the chromosomes in mitosis (arrow) (Figures 7A, lower panels). At cytokinesis, control cells exhibited distribution of lamin B1 at both the reforming nuclear envelope surrounding the chromosomes, and midbody, where it co-localizes with α-tubulin (arrow) (Figure 7B, upper panels), while Dp71-depleted cells displayed a drastic reduction of lamin B1 staining in both of these structures (Figure 7B, lower panels). Interesting, we routinely observed a higher proportion of cells at cytokinesis in Dp71-knockdown cell cultures (arrows) than in those of control cells (∼3∶1 ratio), which might indicate a failure of mutant cells to complete cytokinesis.

## Discussion

Previously, by characterization of Dp71-depleted PC12 cells obtained by antisense treatment, we established the participation of dystrophin Dp71 in NGF-based neuronal differentiation and adhesion [15], [16], [17]. In this study, we characterized in further detail a novel phenotype of the Dp71-depleted cells, which is their decreased growth rate. First, we confirmed the altered proliferation of Dp71-depleted cells by different techniques, including standard hemocytometer counting, BrdU incorporation, and MTT assays. Furthermore, we showed by Annexin V-staining assay that the cell growth deficiency of these cells is not related with apoptosis, indicating a cell cycle defect as a feasible cause. Flow cytometry-based analysis of the cell cycle in asynchronic cell cultures revealed a subtle but reproducible increase in the G0/G1 phase of Dp71-depleted cells compared with control cells. Consistent with this, the cell cycle progress of cell cultures released from serum starvation-induced G0/G1 block showed a significantly increased accumulation of Dp71-depleted cells in G0/G1 compared with control cells. These findings suggest a delay in the transition of Dp71-depleted cells from G0/G1 to S, or that a fraction of these cells is unable to exit G0/G1.

Unexpectedly attempts to arrest Dp71-depleted cells in G2/M by nocodazole exposure caused an increased incidence of cells with <2n DNA content, presumably apoptotic, which was absent in the control cells. Mitotic cell death occurs in response to anti-mitotic drugs, and a mechanism that might underlie this phenomenon has been recently proposed [22], [23], [24], [29]. These authors postulate that cell fate is determined by two competing networks that function in opposite directions during mitotic arrest, one that involves activation of the caspase-dependent cell death pathway and another that controls degradation of cyclin B1, thus, exit mitosis (mitotic slippage). Therefore, if cyclin B1 levels fall below the mitotic-exit threshold first, slippage occurs. If the death threshold is breached first, the cell dies in mitosis. Thus, it appears that Dp71-deficient cells exposed to nocodazole are unable to adapt to the spindle assembly checkpoint before dying by apoptosis. Consistent with this, we found no substantial differences in cyclin B1 levels between control and Dp71-depleted cells released from nocodazole-induced G2/M block. During mitotic arrest caused by microtubule-inhibiting drugs, transcription is inhibited [30], [31]; thus, it has been proposed that absence of transcription in mitotic-arrested cells can trigger apoptosis by depletion of short-lived, anti-apoptotic proteins such as cIAP-2, Mcl-1, and FLIP [32], [33], [34].

Abnormal behavior of Dp71-depleted cells during nocodazole-induced mitotic arrest prompted us to analyze the distribution of Dp71 in cells undergoing mitosis and cytokinesis. Interestingly, we found that Dp71 was targeted to mitotic spindle where it was spatially associated with tubulin, as well as to cleavage furrow and midbody in cytokinesis, where it co-localized with actin and tubulin, respectively. The staining of Dp71 in these particular structures appears to be specific because it was drastically reduced in Dp71-depleted cells. As an attempt to sight into the function of Dp71 in PC12 cell division, we analyzed the effect of Dp71 downregulation on lamin B1 expression; because this protein is an structural component of the spindle matrix implicated in microtubule assembly and organization in mitosis [26], [27], [35] and was found associated with Dp71 in the nuclei of HeLa cells in our previous work [28]. Thus, we envisaged that Dp71 may participate in PC12 mitosis through its binding to lamin B1. Consistent with this hypothesis, we revealed by immunoprecipitation and pull-down assays that lamin B1 binds to Dp71 in PC12 cells. Furthermore, lamin B1 localizes to mitotic spindle and midbody in cells at cytokinesis, cell areas where Dp71 was also targeted. It is noteworthy that Dp71-depleted cells exhibited decreased lamin B1 total protein levels and reduced immunostaining of this protein in mitotic spindle and midbody, which implies that Dp71 and lamin B1 could be functionally linked during cell division. It has been recently revealed that HeLa cells with reduced expression of lamin B1 exhibited complex phenotypes, including disorganized mitotic spindles, presence of binucleated cells, altered chromatin structure, deterioration in nuclear compartmentalization, and apoptosis [27], which evidences that lamin B1, aside from its role supporting the nuclear envelope and providing anchorage sites for chromatin [36], [37], [38], [39], [40], [41], it is involved in chromosome segregation and post-mitotic nuclear assembly. Although we did not found visible alterations in the mitotic spindle morphology of Dp71-depleted cells, it is not precluded that decreased levels of lamin B1 due to down regulation of Dp71 expression could provoke a subtle but functionally important disruption in the mitosis of these cells; for instance, a delay in chromosome alignment and segregation, as reported for HeLa cells with lamin B1 deficit [26]. To test this hypothesis, control and Dp71-depleted cells released from serum starvation-mediated G0/G1 arrest would be subjected to time lapse imaging by using a differential interference contrast microscopy and a temperature-controlled stage, in order to monitor progression of mitosis and quantified the time elapsed from the first sign of chromosome alignment to chromosome separation in individual cells.

On the other hand, it appears that reduced levels of lamin B1 are related with abnormal accumulation of Dp71-depleted cells in G0/G1 cell cycle phases. At the onset of mitosis, lamins are phosphorylated by Cdk1, which leads to the disassembly of nuclear lamin [42], [43], [44], and during post-mitotic nuclear assembly, the majority of the B-type lamin remain attached with membrane vesicles and thus associate with the nuclear periphery during membrane deposition, whereas the bulk of A-type lamin is imported through nuclear pores into the new assembly nucleus and is incorporated into the lamina [45]. As nuclear reassembly begins during the late stages of mitosis and continues during the early stages of G1 phase of the cell cycle [27], it is feasible to propose that low levels of lamin B1 alter the nuclear assembly and nuclear compartments organization of Dp71-depleted cells, which in turn might result in delayed transition from G1 to S. Supporting this idea, we observed abnormal cytoplasmic accumulation of emerin (nuclear envelope protein), which is indicative of defective nuclear assembly.

In our previous work, we established that Dp71 is crucial for the stability of dystrophin-associated proteins (DAPs) in PC12 cells, in such a way that knockdown of Dp71 expression decreases in consequence the protein levels of different DAPs, including β-dystroglycan [18], it was recently reported that β-dystroglycan is targeted to cleavage furrow and midbody of cells undergoing cytokinesis, and that deficiency of this protein provokes cell cycle alterations and apoptosis, probably by altering the extracellular-related kinase (ERK) signaling pathway [25]. According to this, we found that β-dystroglycan was localized to mitotic spindle, cleavage furrow, and midbody of cells at cytokinesis. Interestingly, distribution of β-dystroglycan in these particular zones was drastically reduced in Dp71-depleted cells, suggesting the participation of β-dystroglycan in the abnormal proliferation phenotype of Dp71-depleted cells. If Dp71 and/or β-dystroglycan were crucial for cytokinesis, then low levels of these proteins might lead to a failure in cytokinesis. Although the lack of multinucleated cells in Dp71-depleted cell cultures would appear to act against this hypothesis, the fact that a higher proportion of cells undergoing cytokinesis were observed in Dp71-depleted cell cultures might indicate a delay in this process. To approach this issue, lived cultures of control and Dp71-depleted cells stably transfected with GFP-β-dystroglycan (marker of the cleavage furrow and midbody) and released from G0/G1 arrest, would be subjected to time-lapse fluorescent microscopy analysis, in order to track individual cells undergoing mitosis and record the elapsed time at cytokinesis.

In the light of our results and taking into consideration the well-characterized function of Dp71 as the scaffolding protein involved in the clustering of a number of protein-complexes at cell membrane and cytoskeleton, we propose that Dp71 is a component of the multi-protein apparatus of mitosis and cytokinesis that might participate in their assembly and/or maintenance, conferring stability to some of their components, such as lamin B1 and β-dystroglycan. Furthermore, as nocodazole interferes with cell-cycle checkpoints by intereacting with the spindle assembly machinery, it is tempting to hypothesize that mitotic death underwent by Dp71-depleted cells may have been due to deficiency of lamin B1, β-dystroglycan and Dp71 levels, which might cause mitotic spindle instability, and ultimately high sensitivity of these cells to nocodazole exposure.

## Supporting Information

Figure S1 **Altered proliferation of Dp71-knockdown cells is not related to apoptosis.** Control and Dp71-knockdown cells (AS1) were cultured in normal conditions for 48 h, then cells were harvested, washed, and stained with both annexin-V-FITC and propidium iodide (PI) to measure early and late apoptosis by flow cytometry.
(TIF)

Figure S2 **S phase transition of control and Dp71-depleted cells.** Control and Dp71-depleted (AS1) cells were synchronized at S phase by double thymidine treatment (time 0) and then released into the cell cycle for the indicated time periods. Cell cycle profiles of fixed cells were analyzed by flow cytometry, and their graphical representation is shown. Data are the mean ± standard deviation (SD) of three independent experiments.
(TIF)

Figure S3 **Effect of Dp71-knockdown expression on the subcellular distribution of emerin and lamin A/C.** Control and Dp71-antisense (AS1 clone) cell cultures were fractionated into Total (T), Cytoplasmic (C), and Nuclear (N) protein extracts, and equal amounts of each extract (50 µg) were resolved by SDS-PAGE and subjected to western blotting analysis using antibodies directed to lamin A/C and emerin. As loading control, membranes were stripped and reproved with an anti-actin antibody. *Overexposed membrane. Position of protein markers is shown on the left.
(TIF)
