# Supplementary figures and images for: Knockdown of Dystrophin Dp71 Impairs PC12 Cells Cycle: Localization in the Spindle and Cytokinesis Structures Implies a Role for Dp71 in Cell Division
Source: PLoS One. 2011 Aug 19;6(8):e23504. doi: 10.1371/journal.pone.0023504 (PMC3158767; doi:10.1371/journal.pone.0023504)

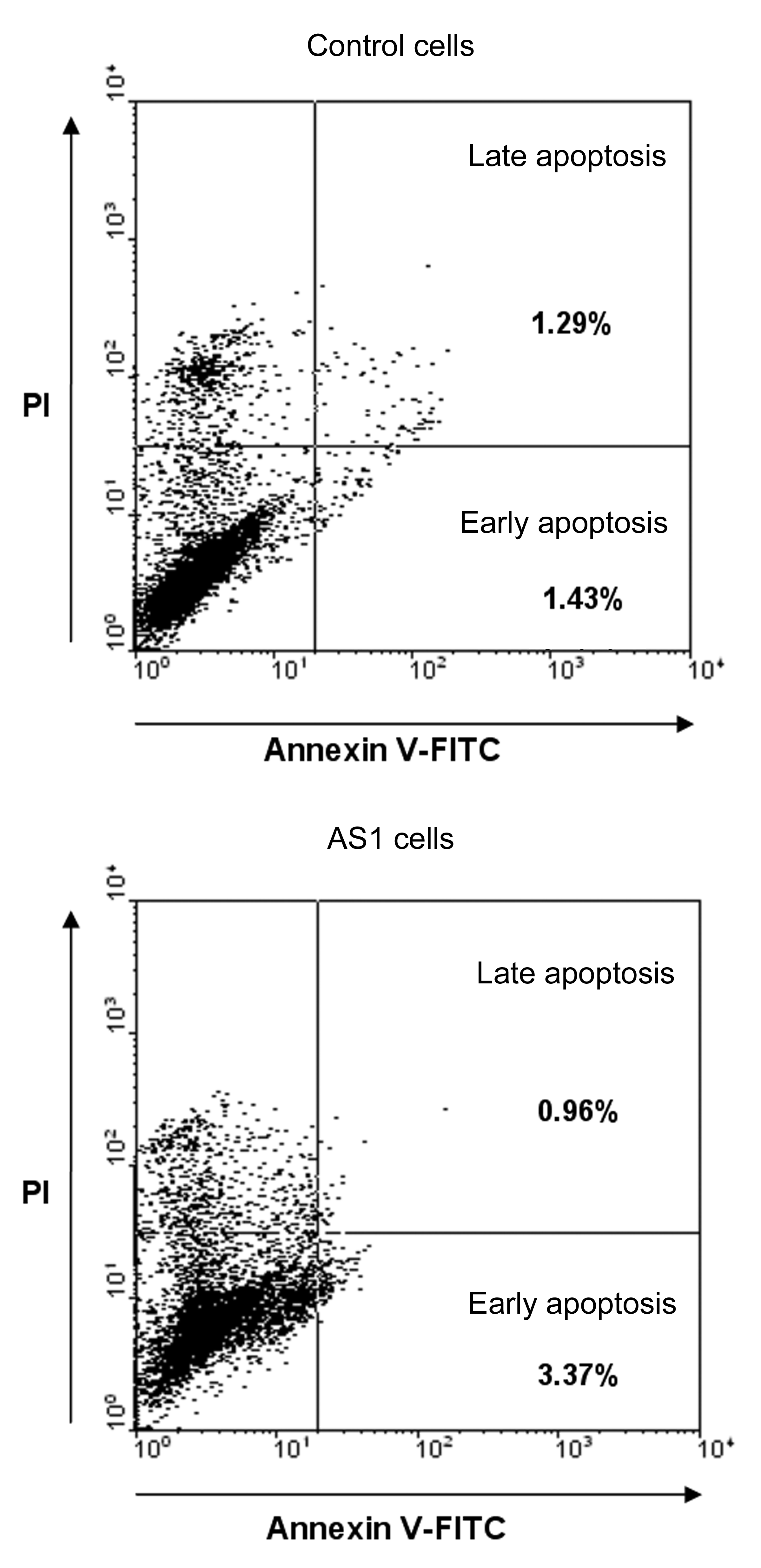

Supplement: Figure S1 — Altered proliferation of Dp71-knockdown cells is not related to apoptosis. Control and Dp71-knockdown cells (AS1) were cultured in normal conditions for 48 h, then cells were harvested, washed, and stained with both annexin-V-FITC and propidium iodide (PI) to measure early and late apoptosis by flow cytometry. (TIF) [file pone.0023504.s001.tif]

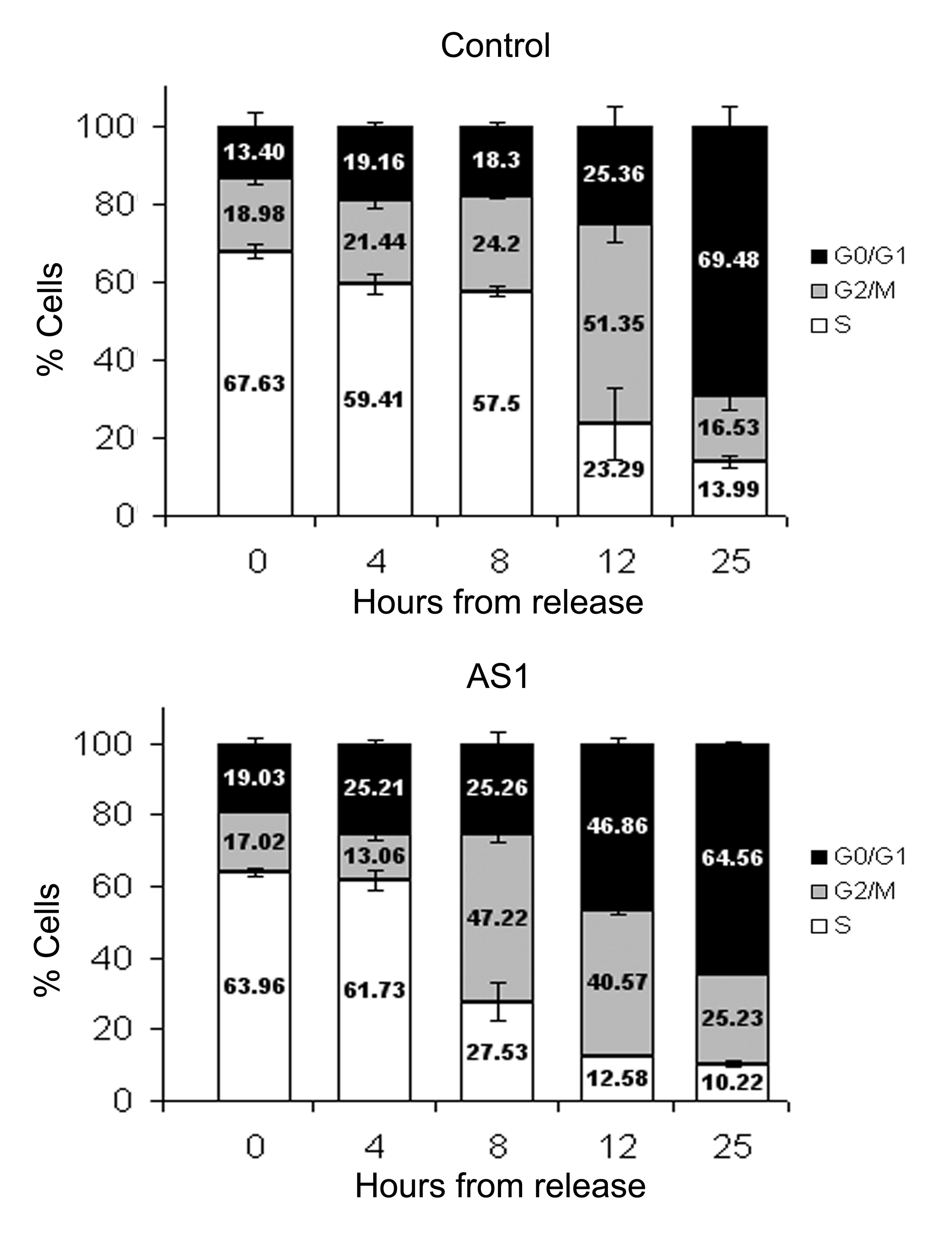

Supplement: Figure S2 — S phase transition of control and Dp71-depleted cells. Control and Dp71-depleted (AS1) cells were synchronized at S phase by double thymidine treatment (time 0) and then released into the cell cycle for the indicated time periods. Cell cycle profiles of fixed cells were analyzed by flow cytometry, and their graphical representation is shown. Data are the mean ± standard deviation (SD) of three independent experiments. (TIF) [file pone.0023504.s002.tif]

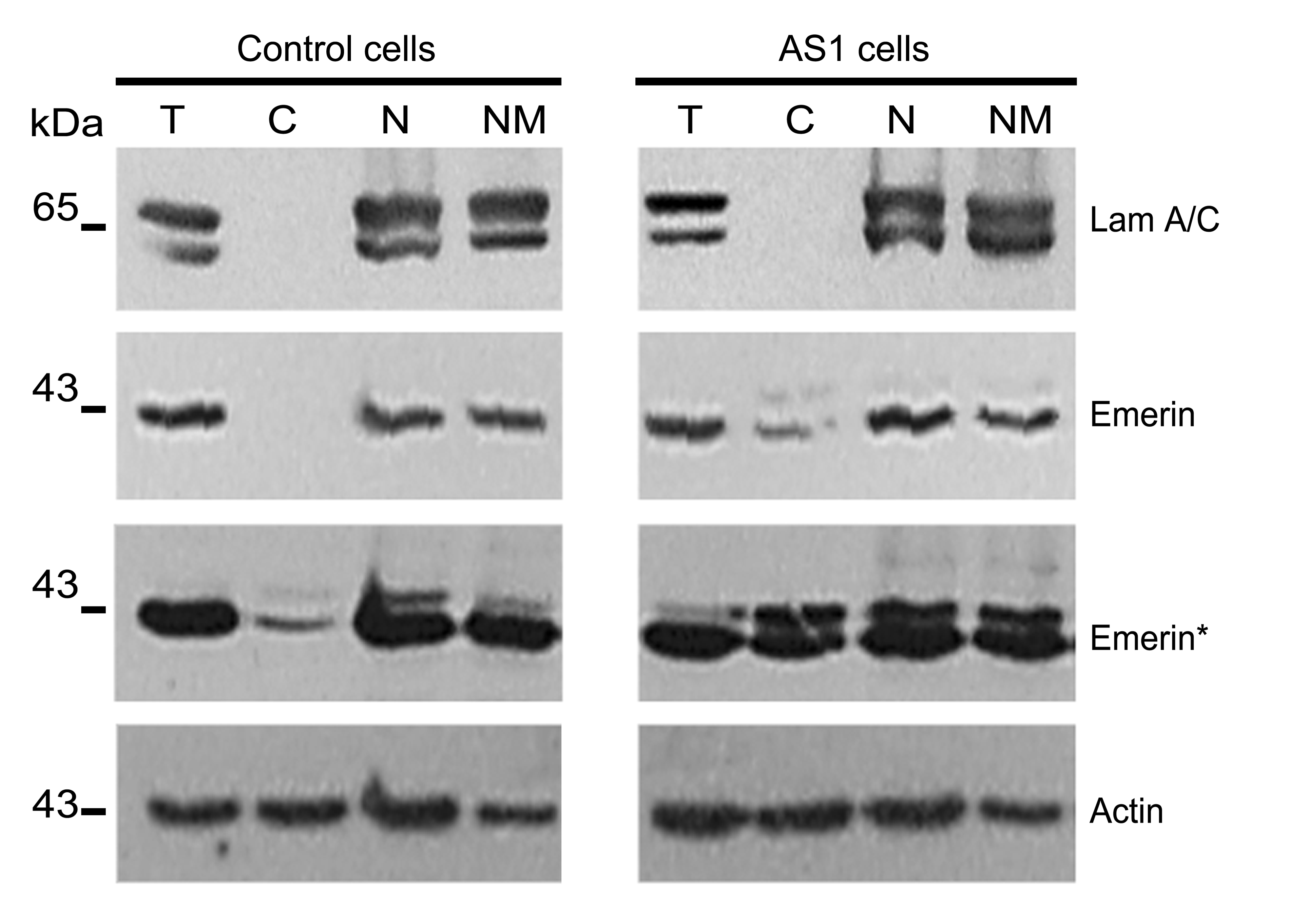

Supplement: Figure S3 — Effect of Dp71-knockdown expression on the subcellular distribution of emerin and lamin A/C. Control and Dp71-antisense (AS1 clone) cell cultures were fractionated into Total (T), Cytoplasmic (C), and Nuclear (N) protein extracts, and equal amounts of each extract (50 µg) were resolved by SDS-PAGE and subjected to western blotting analysis using antibodies directed to lamin A/C and emerin. As loading control, membranes were stripped and reproved with an anti-actin antibody. *Overexposed membrane. Position of protein markers is shown on the left. (TIF) [file pone.0023504.s003.tif]
